# Expression status of circ-SMARCA5, circ-NOL10, circ-LDLRAD3, and circ-RHOT1 in patients with colorectal cancer

**DOI:** 10.1038/s41598-023-40358-4

**Published:** 2023-08-16

**Authors:** Neveen A. Hussein, Shehata M. El Sewedy, Mohamed M. Zakareya, Engy A. Youssef, Fawziya A. R. Ibrahim

**Affiliations:** 1 Applied Medical Chemistry Department, Medical Research Institute, Alexandria University, Alexandria, Egypt; 2 Colorectal Surgical Unit, Faculty of Medicine, Alexandria University, Alexandria, Egypt

**Keywords:** Biochemistry, Cancer, Biomarkers

## Abstract

Colorectal cancer (CRC) poses a significant burden on both the healthcare systems as well as individuals. The high mortality rate of CRC may be attributed to its metastatic potential, heterogeneity, and delayed diagnosis. CircRNAs are an essential class of regulatory RNAs that play significant roles in cancers. This study aimed to detect the expression status of circ-SMARCA5, circ-NOL10, circ-LDLRAD3, and circ-RHOT1 in patients with CRC. This study included 50 CRC patients, 30 individuals with colorectal diseases (non-cancer), and 20 healthy volunteers. By using real-time PCR, the relative expression of circ-SMARCA5, circ-NOL10, circ-LDLRAD3, and circ-RHOT1 was determined in the collected blood samples. In addition, ECLIA was used to quantify carcinoembryonic antigen (CEA) level. All circRNAs expression and CEA levels were significantly up-regulated in cancer patients (CRC, colon, rectum) as compared to healthy controls, except circ-SMARCA5. Moreover, there was a significant up-regulation of circRNAs in most non-cancer patients (UC, polyp, piles). Insignificant upregulation was observed in circRNAs and CEA when comparing cancer with non-cancer patients. No correlations were found between the studied parameters and most clinicopathological characteristics of cancer and non-cancer patients. Circ-SMARCA5, circ-NOL10, circ-LDLRAD3, and circ-RHOT1 were differentially expressed in patients with CRC as well as in non-cancer patients. Circ-SMARCA5 and circ-NOL10 may act as tumor suppressors, while circ-LDLRAD3 and circ-RHOT1 may be oncogenes. Circ-SMARCA5, circ-NOL10, circ-LDLRAD3, and circ-RHOT1 could be promising markers for the early detection of CRC.

## Introduction

Colorectal cancer (CRC) is one of the most prevalent malignancies worldwide. Global Cancer Statistics 2020 ranks CRC second in terms of mortality with 9.4% (5.8% and 3.4% for colon and rectum cancers respectively), and third in terms of newly identified cases with 10.0% (6.0% and 3.8% for colon and rectum respectively). The developed countries account for more than half of the cases. More particular, the regions of the world with the highest Human Development Index are showing incidence and mortality rates of CRC that are at least twice as high as regions with low indices^1^.

With the quickly evolving of next-generation sequencing technologies, circular RNAs (circRNAs), as an essential category of regulatory RNAs, have been discovered. CircRNAs are a unique class of noncoding RNAs that are endogenous, durable, and plentiful in mammalian cells with high sensitivity and specificity. CircRNAs are covalently closed loops that lack polyadenylated tail, and polarity. They can function as molecular regulars to exert various mediatory activities in many malignancies, such as microRNA sponges, interact with the binding protein, control gene expressions, and manage translation^2^.

CircRNA SWI/SNF-related matrix-associated actin dependent regular of chromatin A5 (circ-SMARCA5, has circ_0001445) is derived from SMARCA5 gene on chromosome 4. Circ-SMARCA5 is associated with the progression of many malignancies including hepatocellular carcinoma, gastric cancer (GC), glioblastoma, breast cancer, multiple myeloma, and osteosarcoma by controlling of several miRNAs that either promote or suppress tumor growth^3^. Circ-SMARCA5 predominantly functions as a miRNA sponge and may be used as a biomarker in various cancers^4^.

Circ-NOL10 (has_circ_0000977) is encoded by NOL10 gene and is located on chr2:10,784,445–10,808,849. Circ-NOL10 has been reported to be related to lung cancer. Circ-NOL10 was primarily found in the nucleus, and has a prominent role in transcriptional regulation^5^.

Circ-LDLRAD3 (hsa_circ_0006988) is an intragenic gene located at chr11: 36248634–36248980. It was recently discovered to be an oncogene in pancreatic cancer. Circ-LDLRAD3 act as a diagnostic biomarker for pancreatic cancer, and its removal suppressed cancer progress^6,7^ Circ-LDLRAD3 was reported to be upregulated in the plasma of GC patients, suggesting that circ-LDLRAD3 may have a role in the development of GC^8^. The RHOT1 gene is spliced to produce circ-RHOT1, which is involved in the growth, migration, invasion, and prevention of apoptosis of different cancers (such as liver, breast, and pancreatic cancers)^9^.

Emerging research suggested that the aberrant expression of circRNAs contributes to the development of cancer, so this study aimed to detect the expression status of circ-SMARCA5, circ-NOL10, circ-LDLRAD3 and circ-RHOT1 in patients with CRC, and to explore their correlations with clinicopathological characteristics of the patients.

## Subjects and methods

A total of 50 CRC patients (32 colon, 18 rectum), 30 patients with colorectal disease as ulcerative colitis (UC), polyp, piles, (10 each, non-cancer patients), and 20 healthy volunteers were enrolled in this study. Cancer and non-cancer patients were collected from those who admitted to EL-Maury Hospital, Colorectal Surgical Unit, Faculty of Medicine, Alexandria University. Patients who were pathologically diagnosed with CRC were included in this study while patients who received adjuvant chemo- and radiotherapy or had a history of other types of malignancy were excluded.

### Ethics statement

A signed informed consent was obtained from all participants and clinical experiments were approved by the Ethics Committee of Medical Research Institute (code: E/C.S/N.4/2020), Alexandria University and in accordance with the Declaration of Helsinki.

### Sample collection

Blood samples were taken from CRC patients preoperatively, non-cancer patients, and controls. In the K_3_EDTA-containing tube, 3 ml of blood were pipetted for relative quantification of circRNAs by real time PCR. In another tube without anticoagulant, the remaining blood (3 ml) was collected. Sera have been utilized for determination of carcinoembryonic antigen protein (CEA) by ECLIA.

### Relative expression of circ-SMARCA5, circ-NOL10, circ-LDLRAD3, and circ-RHOT1

RNeasy® Mini Kit (Qiagen, Germany) was used for the extraction and purification of total blood RNA. For each sample, RNA concentration (absorbance at 260 nm) and purity (A260/A280 ratio) were measured (Nano-Drop spectrophotometer, Thermo Fisher Scientific, USA). High-Capacity cDNA Reverse Transcription Kits (Applied Biosystems, USA) were used to reversely transcribed RNA to cDNA.

The expression of circRNAs and GAPDH as housekeeping gene was determined by real time PCR using SYBR Green master mix (Qiagen). Primers sequences (Invitrogen, Inc, USA) were designed using blast software (https://www.ncbi.nlm.nih.gov). The relative expression was assessed by 2^−ΔΔCt^ method.

**Table:** 

Genes	Primers
Circ-SMARCA5	F: GGAGGCTTGTGGATCAGAATC
	R: TCTCACCTTCTTTGCACCTCT
Circ-NOL10	F: TCCCTTCCTGAGATGTCCGT
	R: GCAAACAAGCCATGCACTGA
Circ-LDLRAD3	F: GCCTGACTGCTTCGACAAGA
	R: RAATGATGCAATGGATGCCGC
Circ-RHOT-1	F: GGGAGGAACCTCTTCTGGA
	R: ATGAAGAAAGACGTGCGGAT
GAPDH	F: GGGAAACTGTGGCGTGAT
	R: GAGTGGGTGTCGCTGTTGA

### Serum CEA

The CEA level (ng/ml) was performed by electrochemiluminescence immunoassay (ECLIA, Roche Diagnostics Gmbh, Germany) using Cobase 411 immunoassay analyzer.

### Statistical analyses

Data were fed to the computer and analyzed using IBM SPSS software package version 20.0. Mann Whitney and Kruskal Wallis tests were used for not normally distributed quantitative variables to compare between two or more groups respectively with Post Hoc (Dunn's multiple comparisons test) was used for pairwise comparisons. Spearman coefficient was used for correlations. The area under the receiver operating characteristic curve (ROC) denotes the diagnostic performance of the test. Area greater than 50% gives an acceptable performance.

## Results

### Characteristics of studied groups

Characteristics of control, cancer (CRC, colon, and rectum), and non-cancer subjects (UC, polyp, and piles) are shown in Tables 1, 2. For CRC, 16 patients had stage I, 7 patients had stage II, 23 patients had stage III and 4 patients had stage IV. Regarding histological grade 9 patients had grade I, 23 patients had grade II, 13 patients had grade III and 5 patients had grade IV.

**Table 1:** Characteristics of control and cancer subjects (CRC, colon, and rectum).

	Control n = 20 (%)	Cancer patients		
		CRC n = 50 (%)	Colon cancer n = 32 (%)	Rectum cancer n = 18(%)
Sex				
Female	10 (50)	20 (40)	13 (40.6)	7 (38.8)
Male	10 (50)	30 (60)	19 (59.4)	11 (61.2)
Age				
≥ 60	6 (30)	26 (52)	20 (62.5)	6 (33.3)
< 60	14 (70)	24 (48)	12 (37.5)	12 (66.7)
Smoking				
Yes	10 (50)	30 (60)	19 (59.4)	11 (61.2)
No	10 (50)	20 (40)	13 (40.6)	7 (38.8)
Family history				
Present		2 (4)	2 (6.3)	
Absent		48 (96)	30 (93.7)	18 (100)
Diabetes mellitus				
Present		14 (28)	11 (34.4)	3 (16.7)
Absent		36 (72)	21 (65.6)	15 (83.3)
Blood pressure				
Present		10 (20)	6 (18.75)	4 (22.22)
Absent		40 (80)	26 (81.25)	14 (77.78)
Hb (g/dl)				
Female ≤ 12	4 (40)	16 (32)	11 (34.3)	5 (28)
> 12	6 (60)	4 (8)	2 (6.3)	2 (11)
Male ≤ 13	4 (40)	26 (52)	16 (50)	10 (55.5)
> 13	6 (60)	4 (8)	3 (9.4)	1 (5.5)
RBCs (10^6^/µl)				
Female ≤ 3.8	1 (10)	6 (12)	4 (12.6)	2 (11)
> 3.8	9 (90)	14 (28)	9 (28.1)	5 (28)
Male ≤ 4.5	3 (30)	16 (32)	10 (31.2)	6 (33)
> 4.5	7 (70)	14 (28)	9 (28.1)	5 (28)
PLT (10^3^/µl)				
≥ 400	2 (10)	10 (20)	9 (28.2)	1 (5.5)
< 400	18 (90)	40 (80)	23 (71.8)	17 (94.4)
WBCs (10^3^/µl)				
≥ 11		6 (12)	5 (15.6)	1 (5.5)
< 11	20 (100)	44 (88)	27 (84.4)	17 (94.4)
CEA (ng/ml)				
≥ 5		32 (64)	22 (68.7)	10 (55.6)
< 5	20 (100)	18 (36)	10 (31.3)	8 (44.4)
Tumor location				
Right colon		6 (12)	6 (18.75)	
Left colon		24 (48)	24 (75)	
Transverse colon		2 (4)	2 (6.25)	
Rectum		18 (36)		18 (100)
Tumor size				
≥ 4		17 (34)	10 (31.3)	7 (38.8)
< 4		33 (66)	22 (68.7)	11 (61.2)
Tumor				
T1		8 (16)	6 (18.7)	2 (11.2)
T2		17 (34)	13 (40.6)	4 (22.2)
T3		20 (40)	9 (28.2)	11 (61.1)
T4		5 (10)	4 (12.5)	1 (5.5)
Lymph nodes				
Present		28 (56)	18 (56.2)	10 (55.6)
Absent		22 (44)	14 (43.8)	8 (44.4)
Metastasis				
Present		6 (12)	4 (12.5)	2 (11.2)
Absent		44 (88)	28 (87.5)	16 (88.8)
Stage				
I		16 (32)	12 (37.5)	4 (22.2)
II		7 (14)	4 (12.5)	3 (16.6)
III		23 (46)	14 (43.7)	9 (50)
IV		4 (80)	2 (6.3)	2 (11.2)
Grade				
I		9 (18)	6 (18.7)	3 (16.7)
II		23 (46)	16 (50)	7 (38.8)
III		13 (26)	6 (18.7)	7 (38.8)
IV		5 (10)	4 (12.6)	1 (5.7)

n, number of subjects.

**Table 2:** Clinicopathological characteristics of non-cancerous patients.

	Non-cancerous patients		
	UC n = 10 (%)	Polyp n = 10 (%)	Piles n = 10 (%)
Sex			
Female	3 (30)	4 (40)	4 (40)
Male	7 (70)	6 (60)	6 (60)
Age			
≥ 60	2 (20)	5 (50)	1 (10)
< 60	8 (80)	5 (50)	9 (90)
Smoking			
Yes	3 (30)	6 (60)	6 (60)
No	7 (70)	4 (40)	4 (40)
Family history			
Present		1 (10)	
Absent	10 (100)	9 (90)	10 (100)
Diabetes mellitus			
Present	3 (30)	2 (20)	1 (10)
Absent	7 (70)	8 (80)	9 (90)
Blood pressure			
Present	3 (30)	5 (50)	5 (50)
Absent	7 (70)	5 (50)	5 (50)
CEA (ng/ml)			
≥ 5	2 (20)	4 (8)	1 (10)
< 5	8 (80)	6 (12)	9 (90)
Hb (g/dl)			
Female ≤ 12	3 (30)	4 (40)	3 (30)
> 12			1 (10)
Male ≤ 13	5 (50)	4 (40)	4 (40)
> 13	2 (20)	2 (20)	2 (20)
RBCs (10^6^/µl)			
Female ≤ 3.8		3 (30)	1 (10)
> 3.8	3 (30)	1 (10)	3 (30)
Male ≤ 4.5	3 (30)	3 (30)	3 (30)
> 4.5	4 (40)	3 (30)	3 (30)
PLT (10^3^/µl)			
≥ 400	2 (20)	3 (30)	1 (10)
< 400	8 (80)	7 (70)	9 (90)
WBCs (10^3^/µl)			
≥ 11	2 (20)	1 (10)	1 (10)
< 11	8 (80)	9 (90)	9 (90)
Type of piles			
Internal			4 (40)
External			6 (60)
Type of polyps			
Adenomatous polyp		5 (50)	
Colon polyps		3 (30)	
Familial adenomatous polyposis		1 (10)	
Anal canal polyp		1 (10)	

n, number of patients.

### CircRNA expressions and CEA level

As compared to control group, the expression of circ-NOL10, circ-LDLRAD3, circ-RHOT1, and CEA level in CRC, colon cancer, and rectum cancer patients was significantly upregulated (*P* ˂ 0.001, 0.001, 0.002), while circ-SMARCA5 was insignificantly upregulated (*P* = 0.233, 0.264, 0.280 respectively). Furthermore, up-regulation of all circRNAs and CEA in UC, polyp, and piles was observed except circ-SMARCA5 was down-regulated (*P* = 0.105, 0.522, 0.002).

**Figure 1:**
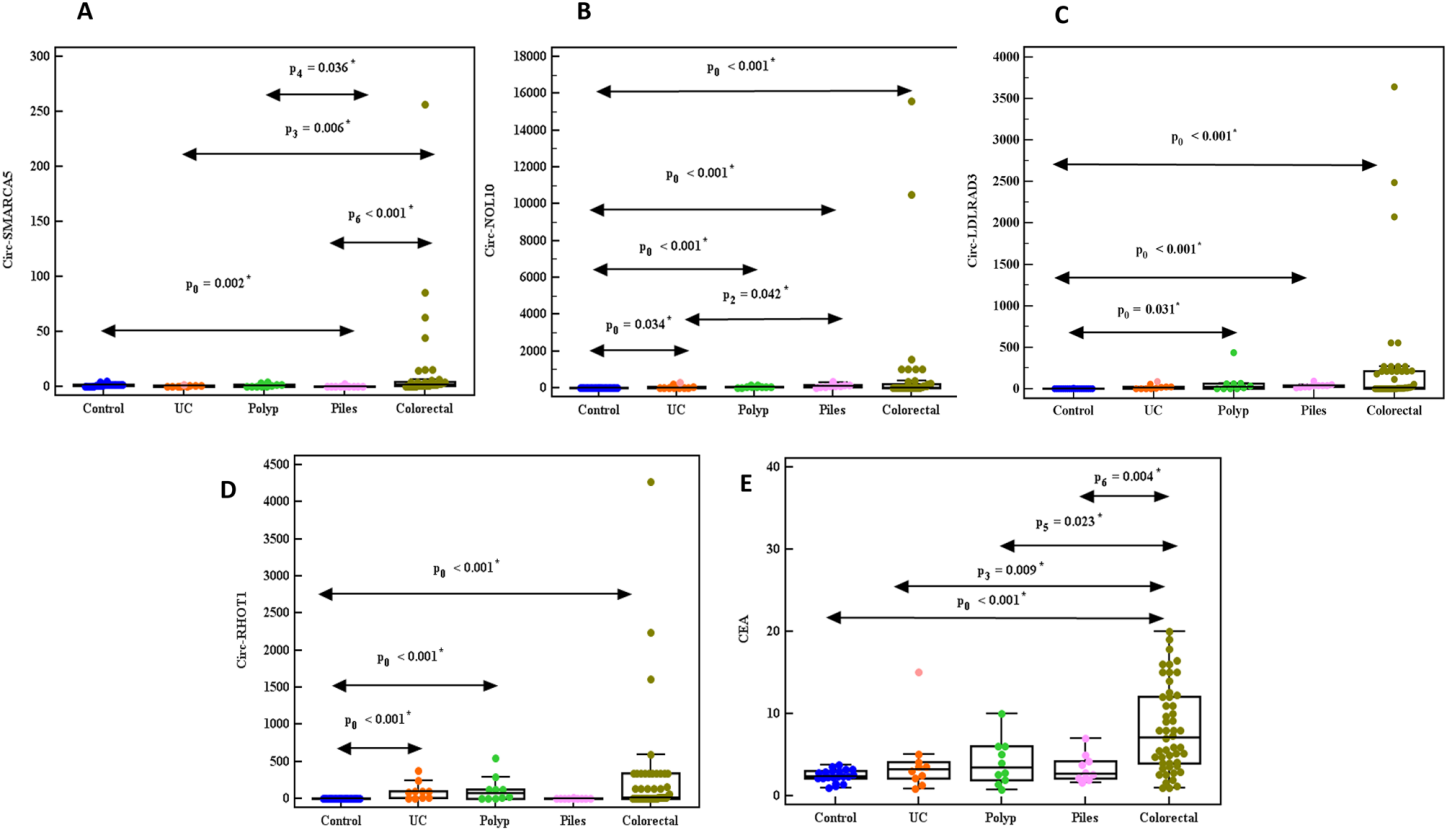
Relative expression of (**A**) circ-SMARCA5, (**B**) circ-NOL10, (**C**) circ-LDLRAD3, (**D**) circ-RHOT1, (**E**) CEA in control, UC, polyp, piles, and CRC groups.

**Figure 2:**
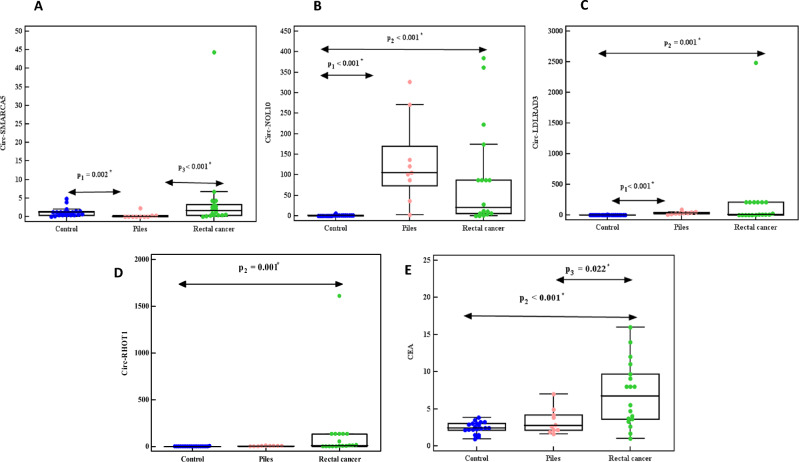
Relative expression of (**A**) circ-SMARCA5, (**B**) circ-NOL10, (**C**) circ-LDLRAD3, (**D**) circ-RHOT1, (**E**) CEA in control, piles, and rectal cancer groups.

**Figure 3:**
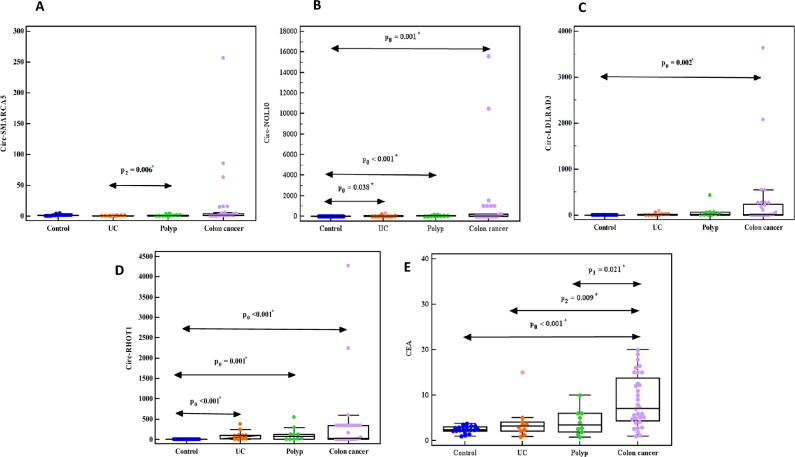
Relative expression of (**A**) circ-SMARCA5, (**B**) circ-NOL10, (**C**) circ-LDLRAD3, (**D**) circ-RHOT1, (**E**) CEA in control, UC, polyp, and colon cancer groups.

The expression of circ-SMARCA5, circ-NOL10, circ-LDLRAD3, circ-RHOT1, and CEA level was up-regulated in CRC compared to UC, polyp, and piles. Also, in colon cancer patients compared to UC, polyp patients as well as in rectum cancer patients compared to piles patients (Figs. 1, 2, 3A–E).

### Correlations between circRNA expressions

Significant positive correlations were found between circ-SMARCA5, circ-NOL10, circ-LDLRAD3, circ-RHOT1, CEA in CRC except between each of circ-SMARCA5, circ-LDLRAD3 and CEA. For UC patients, there were positive correlations between circ-RHOT1 and circ-NOL10, circ-LDLRAD3 (*P* = 0.012, 0.041 respectively) while a negative correlation between circ-SMARCA5 and circ-LDLRAD3 (*P* = 0.011). For polyp patients, there were positive correlations between circ-RHOT1 and circ-NOL10, circ-LDLRAD3, CEA (*P* = 0.002, 0.039, 0.043 respectively). For piles patients, there were positive correlations between each of circ-NOL10, circ-LDLRAD3, circ-RHOT1 (*P* ˂ 0.001, 0.023, 0.011 respectively), while negative correlations between circ-SMARCA5 and circ-LDLRAD3 and circ-RHOT1 (P˂0.001, 0.029) (Supplementary Table 1).

### Correlations of circRNA expressions with patients clinicopathological data

There was no correlation between all studied parameters and clinicopathological data (age, HB, RBCs, PLT, WBCs and tumor size) in CRC, and non-cancer patient, except significant negative correlations between circ-SAMARCA5, circ-NOL10 and PLT in piles (*P* = 0.038, 0.025 respectively), circ-LDLRAD3 and age in CRC (*P* = 0.015), while a positive significant correlation between CEA and PLT in piles (*P* = 0.048). Additionally, there was no relation between studied parameters and other clinicopathological data (sex, smoking, diabetes mellitus, blood pressure) in non-cancer patients (*P* ˃ 0.05), while in CRC patients there were significant relations between circ-SAMARCA5 and blood pressure (*P* = 0.044), circ-RHOT1 and sex, smoking, diabetes mellitus, blood pressure (*P* = 0.042, 0.041, 0.045, 0.034 respectively) and between CEA and sex, smoking, lymph nodes (*P* = 0.003, 0.007,0.030 respectively) (Supplementary Tables 2–6).

### ROC curves of circRNA expressions

**Figure 4:**
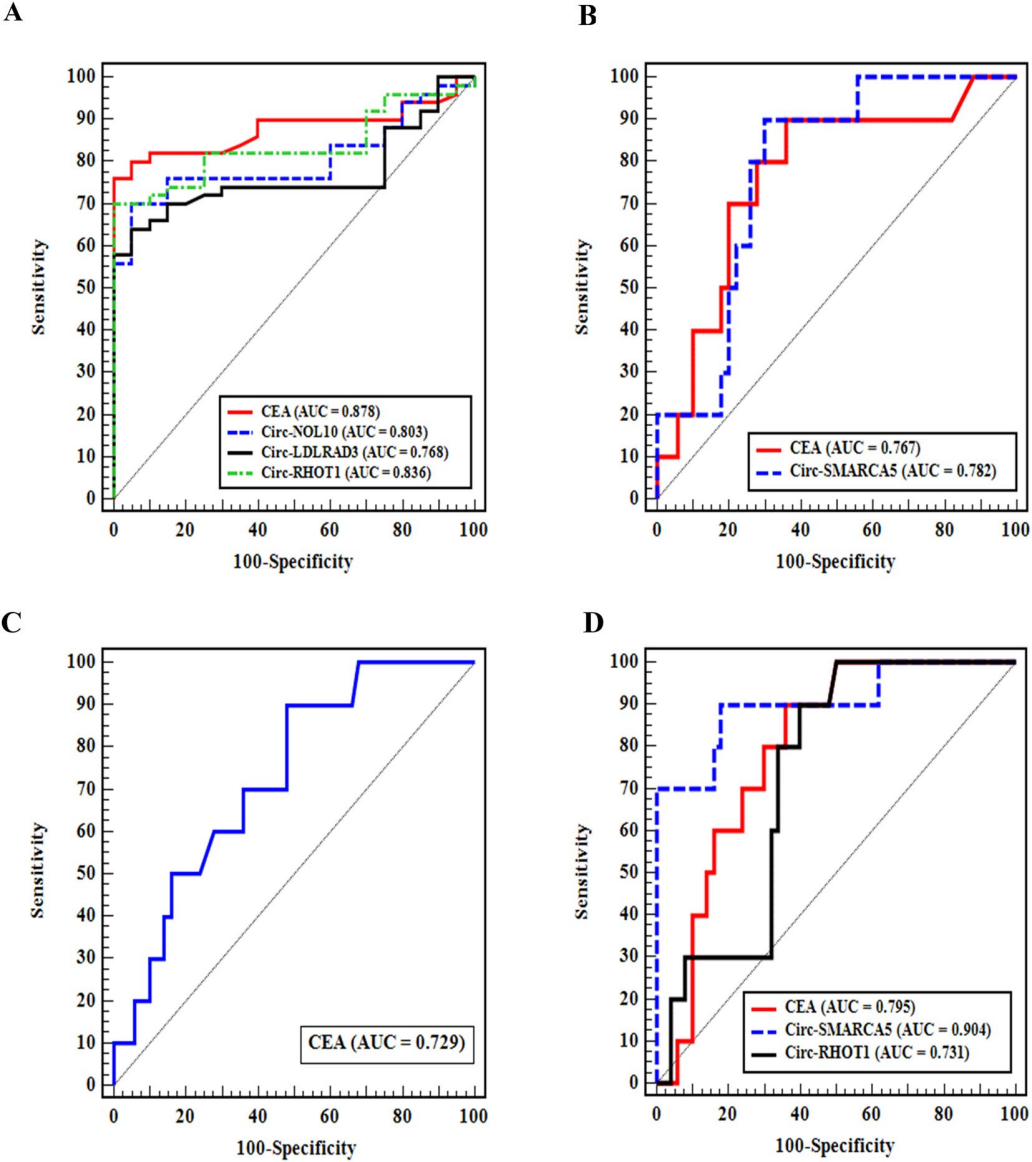
ROC curves for circ-SMARCA5, circ-NOL10, circ-LDLRAD3, circ-RHOT1 and CEA in (**A**) CRC from control, (**B**) UC patients from CRC, (**C**) polyp patients from CRC, and (**D**) piles from CRC.

Based on the area under the curve (AUC), the ROC curve was utilized to evaluate the diagnostic values of the parameters under investigation. A better diagnostic test has a greater AUC. In CRC patients, the sensitivity and specificity of CEA were 76% and 95% respectively, with a substantial AUC (87.8%, *P* < 0.001). The sensitivity and specificity of circ-RHOT1 were 70% and 95% respectively, with a substantial AUC (83.6%, *P* < 0.001). Circ-NOL10 had sensitivity (70%), specificity (95%), and a significant AUC (80.3%, *P* < 0.001). Circ- LDLRAD3 had sensitivity (64%), specificity (95%), and a significant AUC (76.8%, *P* = 0.001). To be differentiated from CRC: in UC patients AUC was considerable, according to circ-SMARCA5 and CEA (*P* = 0.005 and 0.008 respectively). In polyp patients only CEA can be used as a diagnostic marker where AUC was 72.9%, *P* = 0.023. In piles patients a significant AUC was shown by circ-SMARCA5 and circ-RHOT1 (*P* < 0.001 and 0.022 respectively) (Table 3, Fig. 4).

**Table 3:** ROC curve analyses of circ-SMARCA5, circ-NOL10, circ-LDLRAD3, circ-RHOT1 and CEA.

	AUC	P	95% CI	Cut off	Sensitivity	Specificity	PPV	NPV
CRC patients from control								
Circ-SMARCA5	0.611	0.149	0.477–0.745					
Circ-NOL10	0.803	< 0.001*	0.702–0.904	> 1.678^#^	70.0	95.0	97.2	55.9
Circ-LDLRAD3	0.768	0.001*	0.659–0.876	> 2.312^#^	64.00	95.00	97.0	51.4
Circ-RHOT1	0.836	< 0.001*	0.744–0.928	> 2.5847	70.0	95.0	97.2	55.9
CEA	0.878	< 0.001*	0.796–0.959	> 3.7	76.0	95.0	97.4	61.3
UC patients from CRC								
Circ-SMARCA5	0.782	0.005*	0.655–0.909	≤ 0.647^#^	90.0	70.0	37.5	97.2
Circ-NOL10	0.568	0.500	0.381–0.755					
Circ-LDLRAD3	0.560	0.552	0.406–0.714					
Circ-RHOT1	0.558	0.565	0.401	0.715				
CEA	0.767	0.008*	0.606–0.928	≤ 5^#^	90.0	64.0	33.3	97.0
Polyp patients from CRC								
Circ-SMARCA5	0.648	0.142	0.465–0.831					
Circ-NOL10	0.618	0.242	0.478–0.758					
Circ-LDLRAD3	0.540	0.692	0.354–0.726					
Circ-RHOT1	0.506	0.953	0.329–0.683					
CEA	0.729	0.023*	0.576–0.882	≤ 6^#^	90.0	52.0	27.3	96.3
Piles patients from CRC								
Circ-SMARCA5	0.904	< 0.001*	0.783–1.000	≤ 0.28^#^	90.0	82.0	50.0	97.6
Circ-NOL10	0.676	0.081	0.539–0.813					
Circ-LDLRAD3	0.602	0.312	0.469–0.735					
Circ-RHOT1	0.731	0.022*	0.597–0.865	≤ 5.28^#^	90.0	60.0	31.0	96.8
CEA	0.795	0.003*	0.676–0.914	≤ 4.91^#^	90.0	64.0	33.3	97.0

AUC, Area under the curve; *P* value, probability value; CI, confidence intervals; NPV, negative predictive value; PPV, positive predictive value.

*Statistically significant at *P* ≤ 0.05.

^#^Cut off was choosing according to Youden index.

The parameters that showed insignificant differences in cancer or non-cancer patients as compared to the control group or CRC patients respectively could not be utilized as diagnostic markers.

## Discussion

Both the individuals and healthcare systems are significantly burdened by CRC. The heterogeneity, propensity for metastasis, and/or delayed detection of CRC may all contribute to its high mortality rate. CircRNAs have recently been found to play significant roles in several types of cancer. CircRNAs have been shown to control the development of disease because they can bind to miRNAs to control their target genes^10^.

The present results revealed that the expression of all studied circRNAs and CEA level were upregulated in both cancer and non-cancer patients as compared to healthy control except circ-SMARCA5 in non-cancer patients. On the other hand, insignificant upregulation was observed when compared cancer with non-cancer patients.

Up-regulation of circ-SMARCA5 expression was sufficient to impede colon cancer cell viability and change of proteins associated with proliferation (p53, p21, and cyclinD1) and apoptosis (Bax and caspase-3), suggesting that circ-SMARCA5 could suppress the growth of colon cancer. In colon cancer circ-SMARCA5 sponge miR-552, where upregulation of miR-552 partially prevented colon cancer cells from growing and invading. These changes could be linked to the stimulation of signaling pathways in cancer cells. The upregulation of circ-SMARCA5 in colon cancer cells resulted in the inhibition of the Wnt and YAP1 pathways, while miR-552 overexpression restored Wnt and YAP1 pathway activities, suggesting that circ-SMARCA5 had anti-cancer properties^11^.

Furthermore, circ-SMARCA5 could sponge miR-181b-5p and miR-17-3p, which were identified to be onco-miRNAs with proliferative, invasive, and metastatic potential in cancer cells. The tissue inhibitor of metalloproteinases 3 (TIMP3) is a tumor-suppressing substance that, if eliminated, will accelerate tumor invasion and metastasis. MiR-181b-5p or miR-17-3p diminished the inhibitory impact of circ-SMARCA5 on tumor cell, confirming that circ-SMARCA5 could sponge miR-181b-5p, as well as miR-17-3p, to induce TIMP3 and suppress malignant behaviors^12,13^.

Circ-NOL10 had an anticancer impact in many cancer tissues. In lung cancer, circ-NOL10 increases the expression of transcription factor sex comb on midleg‐like 1 (SCML1) by preventing transcription factor ubiquitination and consequently alters the regulation of the humanin polypeptide family by SCML1. Through controlling the humanin polypeptide family and other signaling pathways, Circ-NOL10 also influences mitochondrial activity. As a result, it promotes apoptosis and reduces the growth of cancer cells^5^. Moreover, circ-NOL10 sponges miR-767-5p in breast cancer (BC) to promote the expression of suppressors of cytokine signaling 2 (SOCS2) and attenuate janus kinase 2/signal transducer and activator of transcription 5 (JAK2/STAT5) signaling, therefore preventing cancer progression^14^.

circ-NOL10 upregulation can significantly inhibit the proliferation, progression, and migration of CRC cells. Circ-NOL10 sponge miR-135a-5p and miR-135b-5p to regulate Kruppel-like factor 9 expression, where there was a negative regulatory link between miR-135a/b-5p and KLF9 in CRC^15^.

Circ-LDLRAD3 was an oncogenic factor in non-small cell lung cancer through miR-491-5P / mitogen-activated protein kinase kinase kinase 3 (MAP3K3) and miR-137 / lysine (K)-specific demethylase 1A (SLC1A5) pathways. In vitro, miR-491-5P overexpression prevented cell proliferation, promoted apoptosis and cell cycle arrest. MAP3K3 was identified as a target for miR-491-5p. According to rese arch, MAP3K3 plays an oncogenic role by acting as a target for miRNAs such miR-194, miR-212-3p, miR-188, and miR-4458. Circ-LDLRAD3 may control MAP3K3 expression with miR-491-5p as a crosstalk^16^. The expression of IGF2 at both protein and mRNA levels may be significantly decreased by miR-491-5p overexpression. The tumor-suppressing effects of miR-491-5p on CRC cells might be reversed by upregulating IGF2. Collectively, miR-491-5p exerted its antitumor effect on CRC cells by targeting IGF2^17^.

As a tumor suppressor, MiR-137 stimulates NSCLC apoptosis and prevents its growth and invasion. The downstream target of circ-LDLRAD3 and miR-137 was confirmed to be glutamine transporter solute carrier family A1 member 5 (SLC1A5). SLC1A5 is important for the metabolism of L-glutamine, which necessary for developing cancer cells. MiR-137 served as a post-transcriptional regulator to silence SLC1A5 by targeting its 3ʹ untranslated regions. Circ-LDLRAD3 positively regulated SLC1A5 by sponging miR-137^18^.

Down expression of miR-137-3p might induces KDM1A (also known as lysine-specific demethylase 1) and promote the EMT pathway, which is deeply linked to CRC metastasis^19^. KDM1A expression is frequently increased in various malignancies, and its upregulation is thought to induce cancer cell proliferation, and migration^20^. Since miRNA-491-5P and miR-137-3P are expressed in CRC, circ-LDLRAD3 may be regulate CRC progression by sponging these miRNAs.

Circ-RHOT1 controls the proliferation of pancreatic cells by downregulating the expression of miR-125a-3p to increase E2F3 expression. PANC-1 cells had lower levels of miR-125a-3p, and its overexpression had a negative impact on PANC-1 growth^21^. E2F3 is a transcription factor that belongs to the E2F family and is elevated in several malignancies. It may play a significant role in tumor growth^22^. MiR-125a-3p/E2F3 axis is crucial for PANC-1 cell invasion^21^.

The expression of miR-125a-5p was significantly reduced in CRC tissues and cell lines. In vitro, miR-125a-5p inhibition accelerated the development of CRC. Vascular endothelial growth factor-A (VEGFA) may be a viable target gene of miR-125a-5p, where it could bind to its receptor VEGFR2 and be strongly related to tumor growth. MiR-125a-5p prevented the progression of CRC by VEGFA/VEGFR2 signaling pathway. Mechanisms by which miR-125 controls CRC form a complicated network, miR-125 could negatively modulate B cell lymphoma-2 like protein-12 (BCL2L12), B cell lymphoma-2 (BCL2), and myeloid cell leukemia-1 (Mcl-1), and down-regulated miR-125 promoted colon cancer cell proliferation and suppressed apoptosis^23,24^.

Another mechanism that miR-125 controls CRC development is through controlling signaling pathways. MiR-125 targeted fucosyltransferase 5 and fucosyltransferase 6 and altered the PI3K-AKT signaling pathway, which in turn affected CRC development^25^. The Hippo signaling system, which is critical for cancer cell invasion, regulates transcriptional co-activator with PDZ-binding motif (TAZ) and Yes-associated protein (YAP) by restricting their entry into the nucleus. By targeting TAZ, miR-125 suppress CRC proliferation and invasion^26^.

Moreover, circ-RHOT1 sponges miR-326 that is poorly expressed and has suppressive functions in a variety of malignancies. Pyruvate dehydrogenase kinase 2 (PDK2) is essential for cancer's glycolysis and promotes tumor growth. Circ-RHOT1 may act as a target for miR-326 to indirectly control PDK2 expression and therefore affect cancer development, whereas circ-RHOT1 positively controlled PDK2 expression by sponging miR-326^27^.

In CRC, miR-326 has ability to impede cancer growth by directly inhibiting E2F1. Up-regulation of E2F1 can promote CRC proliferation and prevent apoptosis^28^. E2F1 is a crucial transcription factor that involved in S-phase specific expression of ribonucleotide reductase small subunit M2 (RRM2) under physiological situations. On the other hand, in response to DNA damage, E2F1 also triggered RRM2 transcription via ATM/ATR-CHK1 signal pathway. E2F1 regulates the transcription of several genes involved in DNA synthesis, namely thymidine kinase, dihydrofolate reductase, and thymidylate synthase^29^. In CRC cell lines, overexpression of E2F1 upregulated RRM2 expression through enhancing its transcriptional activation^30^.

Circ-RHOT1 acts as a molecular sponge for miR-3666. Overexpression of miR-3666 may inhibit proliferative activity, and natural killer cell sensitivity of BC. MiR-3666 may target SMAD5 and negatively regulate SMAD5 expression in BC cells, indicating that SMAD5 is necessary for circ-RHOT1 to exhibit its functional role in the progression of BC^31^. MiR-3666 suppresses the growth and metastasis of CRC cells, and its direct target gene is SATB2. Via the Wnt pathway, the transcription factor SATB2 promoted the development of CRC. MiR-3666 and SATB2 in CRC were negatively correlated^32^.

Since circ-RHOT-1 sponge miR-125a, miR-326 and miR-3666 and these miRNAs are expressed in CRC. So, circ-RHOT-1 may regulate the proliferation and invasion of CRC by sponging these miRNAs.

The elevated level of CEA in CRC patients may be associated with the extent and severity of active inflammation of the colonic mucosa. CEA is expressed in the inflamed mucosa related to hemorrhoidal disease (piles) and in ulcerative colitis patients, but not in healthy mucosa. Furthermore, most adenocarcinomas of the colon, rectum, pancreas, and stomach, as well as breast cancers and NSLC, have overexpressed CEA^33^. CEA proteins are bound to a cell membrane through a glycosylphosphatidylinositol (GPI) anchor linkage. The detected elevation of CEA may be caused by phospholipase enzymes that interfere with the breakdown of GPI binding and the removal of CEA from the cell surface. In addition, during colon cancer progresses, activation of oncogenic c-Ki-ras proteins causes an increase in CEA level and a disturbance of basolateral polarity^34^.

Gastrointestinal tract is affected by inflammatory bowel disease (IBD), a chronic inflammatory disorder that is linked to several pathogenic causes, including immunological, microbial, genetic, and environmental variables. Inflammatory factors have been found to interact with genetic components, like mRNAs and microRNAs, to cause the progression of IBD. CircRNAs exhibit variable expression in UC, and several of them exhibit severe dysregulation. CircRNAs also react to different pro-inflammatory stimuli. Due to their capacity to control gene expression, circRNAs may have changed in concentration^35^. Furthermore, circRNAs are involved in the intestinal epithelial barrier and immune homeostasis disruption, which is a key mechanism to develop IBD^36^.

The microbial communities within polyps and the infiltrating T lymphocytes of polyps regulate intra-polyp inflammatory reactions, which are the primary cause of polyposis. CircRNAs are linked to the abnormal development of inflammatory polyps^37^.

Piles are prevalent disorders that impair the normal functions of the anus and rectum. The vascular wall and the connective tissue around the hemorrhoids suffer from a significant inflammatory response that gradually results in mucosal ischemia, ulceration, and thrombosis. In this region, tissue injury triggers an inflammatory response that emerges as the invasion of neutrophils, macrophages, T lymphocytes, monocytes, and dendritic cells. Both pro- and anti-inflammatory cytokines are secreted by these cells. But because pro-inflammatory cytokines are produced in excess, the pro-inflammatory response eventually dominates the anti-inflammatory response^38^. Upregulated circRNAs were prevalent in anorectal malformations (ARMs) and may have helped ARMs develop, where they have an impact on cellular, regulatory, and metabolic processes^39^.

Lack of significant correlation between studied parameters and most clinicopathological data may be due to the small number of available samples for patients (cancer and non-cancer). These observed results are in line with previous studies which indicated that no significant correlation between circ-SMARCA5 and gender, age, smoking, tumor size, TNM system^40^ and between circ-NOL10 and age, gender, smoking, tumor location^41^ as well as between circ-RHOT1 and tumor size, stage^21^.

In CRC, CEA, circ-RHOT1, circ-NOL10 and circ-LDLRAD3 could be used as diagnostic markers where CEA was superior to the expression of circ-RHOT1 followed by circ-NOL10 and circ-LDLRAD3. To be differentiated from CRC, in UC patients both circ-SMARCA5 and CEA could be used as diagnostic markers where circ-SMARCA5 superior to CEA; in polyp patients CEA could be a diagnostic marker only, and in piles patients circ-SMARCA5 superior to CEA followed by circ-RHOT1 for prediction of piles.

## Conclusion

This study provides new insights into the possible implication of circ-SMARCA5, circ-NOL10, circ-LDLRAD3, circ-RHOT1 in CRC progression, where they are expressed differentially in CRC patients as well as non-cancer patients (UC, polyp, and piles). Since circ-SMARCA5 and circ-NOL10 are tumor suppressor genes, whereas circ-LDLRAD3 and circ-RHOT1 are oncogenes.

Due to the dynamic variation of the expressions of circ-SMARCA5, circ-NOL10, circ-LDLRAD3 and circ-RHOT in cancer, and non-cancer patients, these circRNAs might be prospective candidates for early diagnosis of CRC. However, to validate such a role of the studied circRNAs, large clinical cohort studies are highly recommended.

### Supplementary Information

Supplementary Tables.

## Data Availability

All data generated or analyzed during this study are included in this published article.
