# Supplementary material for: Expression status of circ-SMARCA5, circ-NOL10, circ-LDLRAD3, and circ-RHOT1 in patients with colorectal cancer
Source: Sci Rep. 2023 Aug 16;13:13308. doi: 10.1038/s41598-023-40358-4 (PMC10432413; doi:10.1038/s41598-023-40358-4)
Supplement: Supplementary file 1 — Supplementary Tables. [file 41598_2023_40358_MOESM1_ESM.docx]

**Table (1): Correlation between circ-SMARCA5, circ-NOL10, circ-LDLRAD3, circ-RHOT1 and CEA**

|  |  | **CRC** | | | | **UC** | | | | **Polyp** | | | | **Piles** | | | |
| --- | --- | --- | --- | --- | --- | --- | --- | --- | --- | --- | --- | --- | --- | --- | --- | --- | --- |
|  |  | **circ-NOL10** | **circ-LDLRAD3** | **circ-RHOT1** | **CEA** | **circ-NOL10** | **circ-LDLRAD3** | **circ-RHOT1** | **CEA** | **circ-NOL10** | **circ-LDLRAD3** | **circ-RHOT1** | **CEA** | **circ-NOL10** | **circ-LDLRAD3** | **circ-RHOT1** | **CEA** |
| **Circ-SMARCA5** | r_s_ | 0.238 | -0.117 | 0.261 | 0.141 | 0.261 | -0.455 | -0.279 | -0.258 | 0.113 | -0.164 | -0.027 | 0.175 | -0.292 | -0.718 | -0.398 | -0.030 |
|  | P | 0.047^*^ | 0.334 | 0.029* | 0.244 | 0.164 | 0.011^*^ | 0.135 | 0.169 | 0.551 | 0.386 | 0.888 | 0.356 | 0.117 | <0.001^*^ | 0.029* | 0.876 |
| **Circ-NOL10** | r_s_ |  | 0.402 | 0.405 | 0.354 |  | 0.268 | 0.451 | -0.220 |  | 0.347 | 0.547 | 0.160 |  | 0.677 | 0.415 | 0.048 |
|  | P |  | 0.001^*^ | 0.001* | 0.003* |  | 0.153 | 0.012* | 0.242 |  | 0.061 | 0.002* | 0.397 |  | <0.001^*^ | 0.023* | 0.802 |
| **Circ-LDLRAD3** | r_s_ |  |  | 0.242 | 0.199 |  |  | 0.376 | 0.129 |  |  | 0.380 | -0.204 |  |  | 0.458 | -0.074 |
|  | P |  |  | 0.043* | 0.099 |  |  | 0.041* | 0.495 |  |  | 0.039* | 0.279 |  |  | 0.011* | 0.699 |
| **Circ-RHOT1** | r_s_ |  |  |  | 0.327 |  |  |  | 0.200 |  |  |  | 0.372 |  |  |  | 0.153 |
|  | P |  |  |  | 0.006* |  |  |  | 0.289 |  |  |  | 0.043* |  |  |  | 0.421 |

r_s_: Spearman coefficient *: Statistically significant at P ≤ 0.05

**Table (2): Correlation between circ-SMARCA5, circ-NOL10, circ-LDLRAD3, circ-RHOT1, CEA and clinicopathological data (age, HB, RBCs, PLT, WBCs, and tumor size) in CRC, UC, polyp, and piles groups.**

|  | **Circ-SMARCA5** | | **Circ-NOL10** | | **Circ-LDLRAD3** | | **Circ-RHOT1** | | **CEA** | |
| --- | --- | --- | --- | --- | --- | --- | --- | --- | --- | --- |
|  | **r_s_** | **P** | **r_s_** | **P** | **r_s_** | **P** | **r_s_** | **P** | **r_s_** | **P** |
| **CRC** | | | | | | | | | | |
| **Age (years)** | -0.158 | 0.274 | -0.096 | 0.508 | -0.342 | 0.015^*^ | 0.178 | 0.217 | -0.020 | 0.888 |
| **Hb** | 0.220 | 0.124 | -0.028 | 0.846 | -0.243 | 0.088 | 0.269 | 0.059 | -0.082 | 0.573 |
| **RBCs** | 0.085 | 0.555 | -0.114 | 0.432 | -0.248 | 0.082 | 0.269 | 0.059 | -0.071 | 0.625 |
| **PLT** | 0.020 | 0.893 | -0.044 | 0.763 | 0.144 | 0.317 | -0.082 | 0.571 | -0.116 | 0.422 |
| **WBCs** | -0.102 | 0.479 | -0.034 | 0.816 | 0.001 | 0.996 | 0.026 | 0.860 | -0.169 | 0.239 |
| **Tumor size** | -0.048 | 0.739 | -0.215 | 0.134 | -0.059 | 0.683 | 0.055 | 0.704 | 0.239 | 0.094 |
| **UC** | | | | | | | | | | |
| **Age (years)** | 0.064 | 0.861 | 0.085 | 0.815 | -0.436 | 0.208 | -0.280 | 0.432 | 0.445 | 0.197 |
| **Hb** | 0.195 | 0.590 | -0.285 | 0.425 | -0.061 | 0.868 | 0.231 | 0.521 | -0.280 | 0.434 |
| **RBCs** | 0.359 | 0.309 | 0.091 | 0.803 | 0.444 | 0.199 | 0.389 | 0.266 | -0.261 | 0.466 |
| **PLT** | 0.292 | 0.413 | -0.418 | 0.229 | -0.280 | 0.434 | -0.073 | 0.841 | -0.134 | 0.713 |
| **WBCs** | -0.109 | 0.763 | -0.006 | 0.987 | -0.590 | 0.073 | -0.298 | 0.403 | 0.109 | 0.763 |
| **Polyp** | | | | | | | | | | |
| **Age (years)** | 0.256 | 0.475 | 0.312 | 0.380 | -0.006 | 0.987 | 0.272 | 0.448 | 0.394 | 0.259 |
| **Hb** | -0.298 | 0.403 | -0.052 | 0.887 | -0.207 | 0.567 | -0.111 | 0.761 | -0.226 | 0.531 |
| **RBCs** | -0.333 | 0.347 | -0.239 | 0.506 | -0.358 | 0.310 | -0.067 | 0.853 | -0.036 | 0.920 |
| **PLT** | -0.200 | 0.580 | 0.226 | 0.530 | 0.297 | 0.405 | -0.350 | 0.322 | -0.231 | 0.521 |
| **WBCs** | 0.321 | 0.365 | 0.330 | 0.352 | -0.079 | 0.829 | -0.387 | 0.270 | 0.340 | 0.336 |
| **Piles** | | | | | | | | | | |
| **Age (years)** | 0.479 | 0.162 | -0.152 | 0.676 | 0.215 | 0.551 | -0.018 | 0.960 | 0.115 | 0.751 |
| **Hb** | 0.219 | 0.544 | -0.122 | 0.738 | 0.148 | 0.684 | -0.154 | 0.671 | -0.407 | 0.243 |
| **RBCs** | 0.127 | 0.726 | -0.188 | 0.603 | -0.031 | 0.933 | -0.362 | 0.304 | -0.394 | 0.260 |
| **PLT** | -0.661 | 0.038^*^ | -0.697 | 0.025^*^ | -0.129 | 0.723 | -0.190 | 0.599 | 0.636 | 0.048^*^ |
| **WBCs** | 0.139 | 0.701 | -0.103 | 0.777 | 0.055 | 0.880 | -0.337 | 0.340 | 0.055 | 0.881 |

r_s_: Spearman coefficient *: Statistically significant at P ≤ 0.05

**Table (3): Relation of studied parameters with clinicopathological data in CRC group.**

|  | **n** | **Circ-SMARCA5** | **Circ-NOL10** | **Circ-LDLRAD3** | | **Circ-RHOT1** | **CEA** |
| --- | --- | --- | --- | --- | --- | --- | --- |
| **Sex** |  | 6.89±18.83  17.91±57.01 | 573.8±1920.0  829.3±3472.2 | 342.9±847.2  102.7±148.9 | | 381.5±882.5  84.43±135.0 | 6.15±3.69  11.13±5.87 |
| **Male** | 30 |  |  |  |  |  |  |
| **Female** | 20 |  |  |  |  |  |  |
| **U (P)** | | U=246.00 (P=0.285) | U=298.00 (P=0.968) | U=290.00 (P=0.843) | | U=198.0 (P=0.042^*^) | U=150.50 (P=0.003^*^) |
|  | |  |  |  | |  |  |
| **Smoking** |  | 17.07±55.70  7.12±19.12 | 790.3±3389.0  593.2±1951.0 | 98.09±146.7  354.5±859.8 | | 80.82±132.7  394.3±895.3 | 10.79±5.93  6.22±3.74 |
| **No** | 21 |  |  |  |  |  |  |
| **Yes** | 29 |  |  |  |  |  |  |
| **U (P)** | | U=266.00 (P=0.449) | U=302.00 (P=0.961) | U=292.00 (P=0.806) | | U=201.0 (P=0.041^*^) | U=167.0 (P=0.007^*^) |
|  | |  |  |  | |  |  |
| **Family history** |  | 11.70±39.49  1.66±2.25 | 704.2±2673.0  0.48±0.42 | 256.8±681.2  7.36±10.39 | | 273.5±712.4  1.33±0.55 | 8.22±5.34  6.30±0.99 |
| **No** | 48 |  |  |  |  |  |  |
| **Yes** | 2 |  |  |  |  |  |  |
| **U (P)** | | U=36.00 (P=0.589) | U=15.00 (P=0.118) | U=29.00 (P=0.392) | | U=16.00 (P=0.132) | U=44.0 (P=0.864) |
|  | |  |  |  | |  |  |
| **Diabetes mellitus** | | 3.59±7.61  35.69±75.04 | 163.3±358.6  2299.7±5137.9 | 138.3±409.7  590.6±1122.2 | 191.5±690.4  487.8±710.9 | | 8.34±5.14  7.53±5.77 |
| **No** | 38 |  |  |  |  |  |  |
| **Yes** | 12 |  |  |  |  |  |  |
| **U (P)** | | U=215.50 (P=0.776) | U=181.00 (P=0.285) | U=154.50 (P=0.095) | U=140.00 (P=0.045^*^) | | U=205.0 (P=0.601) |
|  | |  |  |  |  | |  |
| **Blood Pressure** |  | 3.35±7.47  43.08±80.74 | 385.5±1661.3  1838.1±4856.2 | 153.2±404.4  621.3±1238.2 | 240.3±747.9  352.2±480.7 | | 8.42±5.44  7.03±4.47 |
| **No** | 40 |  |  |  |  |  |  |
| **Yes** | 10 |  |  |  |  |  |  |
| **U (P)** | | U=117.50^*^ (P=0.044^*^) | U=136.50 (P=0.125) | U=185.50 (P=0.729) | U=113.50 (P=0.034^*^) | | U=174.0 (P=0.542) |
|  | |  |  |  |  | |  |
| **Tumor** |  | 35.67 ± 89.25  6.82 ± 15.25  7.82 ± 20.75  1.43 ± 1.17 | 2270.6±5392.6  734.6 ± 2541.7  152.3 ± 307.3  20.30 ± 37.46 | 324.6 ± 715.3  118.4 ± 187.6  370.6 ± 943.9  63.96 ± 115.3 | | 201.2 ± 230.1  271.4 ± 529.9  262.2 ± 947.1  332.9 ± 712.4 |  |
| **T1** | 8 |  |  |  |  |  | 7.99 ± 5.57  6.78 ± 4.79  8.38 ± 5.02  12.06 ± 6.65 |
| **T2** | 17 |  |  |  |  |  |  |
| **T3** | 20 |  |  |  |  |  |  |
| **T4** | 5 |  |  |  |  |  |  |
| **H (P)** | | H=2.142 (P=0.543) | H=4.568 (P=0.206) | H=1.390 (P=0.708) | | H=2.867 (P=0.413) | H=3.558 (P=0.313) |
|  | |  |  |  | |  |  |
| **Lymph nodes** | |  |  |  | |  |  |
| **Absent**  **Present** | 22  28 | 13.50 ± 54.24  9.56 ± 20.65 | 841.1 ± 3304.3  546.3 ± 1985.2 | 186.2 ± 446.3  294.5 ± 807.4 | | 125.9 ± 172.8  370.1 ± 915.5 | 6.44 ± 4.83  9.48 ± 5.26 |
| **U (P)** | | U=302.0 (P=0.907) | U=298.0 (P=0.845) | U=287.50 (P=0.688) | | U=280.50 (P=0.589) | U=197.0 (P=0.030^*^) |
|  | |  |  |  | |  |  |
| **Metastasis**  **Absent**  **Present** | 44  6 | 12.69 ± 41.14  1.09 ± 1.20 | 757.8 ± 2787.9  76.02 ± 143.9 | 272.7 ± 709.2  56.73 ± 104.4 | | 252.8 ± 714.2  335.0 ± 636.2 | 7.63 ± 5.01  11.93 ± 5.90 |
| **U (P)** | | U=86.0 (P=0.179) | U=114.50 (P=0.610) | U=129.50 (P=0.942) | | U=105.50 (P=0.439) | U=73.0 (P=0.081) |
|  | |  |  |  | |  |  |
| **Stage** | |  |  |  | |  |  |
| **I** | 16 | 18.83 ± 63.41 | 1149.8± 3861.7 | 225.4 ± 517.6 | | 151.7 ± 194.9 | 5.61 ± 4.77 |
| **II** | 7 | 12.36 ± 22.98 | 237.2 ± 579.2 | 62.55 ± 101.7 | | 141.5 ± 147.1 | 9.10 ± 4.44 |
| **III** | 23 | 7.52 ± 19.46 | 577.9 ± 2179.9 | 348.2 ± 884.0 | | 337.2 ± 975.2 | 9.28 ± 5.25 |
| **IV** | 4 | 1.02 ± 1.54 | 112.6 ± 170.7 | 71.84 ± 129.8 | | 489.7 ± 760.6 | 10.05 ± 6.59 |
| **H (P)** | | H=4.991 (P=0.172) | H=0.128 (P=0.988) | H=1.995 (P=0.573) | | H=2.201 (P=0.532) | H=6.857 (P=0.077) |
|  | |  |  |  | |  |  |
| **Grade**  **I**  **II**  **III**  **IV** | 9  23  13  5 | 32.02 ± 84.20  5.14 ± 13.32  11.64 ± 25.22  1.43 ± 1.17 | 2019.4± 5100.4  596.9 ± 2188.8  138.1 ± 280.7  20.30 ± 37.46 | 288.6 ± 677.8  220.0 ± 520.7  335.7 ± 997.2  63.96 ± 115.3 | | 193.5 ± 216.5  186.6 ± 468.6  418.0 ± 1163.4  332.9 ± 712.4 | 7.51 ± 5.40  7.20 ± 4.97  8.75 ± 4.93  12.06 ± 6.65 |
| **H (P)** | | H=1.434 (P=0.698) | H=4.112 (P=0.250) | H=0.672 (P=0.880) | | H=1.494 (P=0.684) | H=3.396 (P=0.334) |
|  | |  |  |  | |  |  |
| **Tumor size** | | 12.33 ± 45.22  9.30 ± 22.28 | 967.1 ± 3199.3  110.9 ± 249.0 | 232.2 ± 551.3  275.2 ± 872.6 | | 183.1 ± 404.6  417.0 ± 1064.8 | 7.19 ± 4.96  10.0 ± 5.44 |
| **<4**  **≥4** | 33 |  |  |  |  |  |  |
|  | 17 |  |  |  |  |  |  |
| **U (P)** | | U=263.50 (P=0.728) | U=247.0 (P=0.492) | U=259.0 (P=0.659) | | U=240.0 (P=0.405) | U=190.0 (P=0.064) |

**Table (4): Relation of studied parameters with clinicopathological data in UC group.**

|  | **n** | **Circ-SMARCA5** | **Circ-NOL10** | **Circ-LDLRAD3** | **Circ-RHOT1** | **CEA** |
| --- | --- | --- | --- | --- | --- | --- |
| **Sex** |  | 0.40 ± 0.20  0.64 ± 0.86 | 54.92 ± 112.5  62.01 ± 99.53 | 26.59 ± 32.46  6.18 ± 9.28 | 105.5 ± 145.3  95.41 ± 7.56 | 4.91 ± 4.60  2.14 ± 1.35 |
| **Male** | 7 |  |  |  |  |  |
| **Female** | 3 |  |  |  |  |  |
| **U (P)** | | U =8.000 (P = 0.667) | U =8.000 (P = 0.667) | U = 5.000 (P = 0.267) | U = 8.500 (P = 0.667) | U = 4.500 (P = 0.183) |
|  | |  |  |  |  |  |
| **Smoking** |  | 0.64 ±0.86  0.40 ±0.20 | 62.01 ± 99.53  54.92 ± 112.5 | 6.18 ± 9.28  26.59 ± 32.46 | 95.41 ± 7.56  105.5 ± 145.6 | 2.14 ± 1.35  4.91 ± 4.60 |
| **No** | 3 |  |  |  |  |  |
| **Yes** | 7 |  |  |  |  |  |
| **U (P)** | | U =8.000 (P = 0.667) | U =8.000 (P = 0.667) | U = 5.000 (P = 0.267) | U = 8.500 (P = 0.667) | U = 4.500 (P = 0.183) |
| **Diabetes Mellitus** | | 0.52 ±0.51  0.34 ±0.32 | 55.45 ± 112.3 | 23.86 ± 33.83  12.53 ± 10.80 | 97.25 ± 127.9  114.6 ± 118.6 | 4.39 ± 4.89  3.37 ± 0.81 |
| **No** | 7 |  |  |  |  |  |
| **Yes** | 3 |  | 60.77 ± 100.6 |  |  |  |
| **U (P)** | U = 8.000 (P = 0.667) | | U = 9.00 (P = 0.833) | U = 8.500 (P = 0.667) | U = 8.500 (P = 0.667) | U = 8.500 (P = 0.667) |
|  |  | |  |  |  |  |
| **Blood Pressure** |  | 0.54 ± 0.51  0.29 ± 0.25 | 12.47 ± 20.35  161.1 ± 153.4 | 26.19 ± 32.80  7.10 ± 8.54 | 78.64 ± 83.15  158.1 ± 190.0 | 4.43 ± 4.78  3.26 ± 1.88 |
| **No** | 7 |  |  |  |  |  |
| **Yes** | 3 |  |  |  |  |  |
| **U (P)** | U = 7.500 (P = 0.517) | | U = 5.00 (P = 0.267) | U = 9.000 (P = 0.833) | U = 8.500 (P = 0.667) | U = 9.500 (P = 0.833) |
|  |  | |  |  |  |  |

The results were expressed mean ± S.D.

U: Mann Whitney test

P: P value for comparing between different categories.

**Table (5): Relation of studied parameters with clinicopathological data in polyp group.**

|  | | **n** | **Circ-SMARCA5** | **Circ-NOL10** | **Circ-LDLRAD3** | **Circ-RHOT1** | **CEA** |
| --- | --- | --- | --- | --- | --- | --- | --- |
| **Sex**  **Male**  **Female** | |  | 0.96 ± 1.65  1.62 ± 1.42 |  | 18.55 ± 26.81  138.0 ± 198.8 |  |  |
|  |  | **6**  **4** |  | 65.71 ± 56.33  82.78 ± 42.27 |  | 135.0 ± 211.1  108.6 ± 129.9 | 3.73 ± 1.56  4.55 ± 4.31 |
| **U (P)** | | | U=5.00 (P=0.171) | U=10.00 (P=0.762) | U=6.00 (P=0.257) | U=11.00 P=0.914) | U=11.5 (P=0.914) |
|  | | |  |  |  |  |  |
| **Smoking** |  | |  |  | 138.0 ± 198.8  18.55 ± 26.81 | 108.6 ± 129.9  135.0 ± 211.1 | 4.55 ± 4.31  3.73 ± 1.56 |
| **No**  **Yes** | | **4**  **6** | 1.62 ± 1.42  0.96 ± 1.65 | 82.78 ± 42.27  65.71 ± 56.33 |  |  |  |
| **U (P)** | | | U=5.000 (P=0.171) | U=10.00 (P=0.762) | U=6.00 (P=0.257) | U=11.00 (P=0.914) | U=11.5 (P=0.914) |
|  | | |  |  |  |  |  |
| **Blood Pressure** | |  | 1.33 ± 1.40  1.13 ± 1.79 | 72.44 ±49.79  72.63 ±54.79 | 98.92 ± 188.7  33.75 ± 28.66 | 62.62 ± 125.2  186.3 ± 208.6 | 4.57 ± 3.47  3.54 ± 2.15 |
| **No**  **Yes** | | **5**  **5** |  |  |  |  |  |
| **U (P)** | | | U=10.00 (P=0.690) | U=12.00 (P=1.000) | U=9.00 (P=0.548) | U=5.00 (P=0.151) | U=10.5 (P=0.690) |
|  | | |  |  |  |  |  |

The results were expressed mean ± S.D.

U: Mann Whitney test

P: P value for comparing between different categories.

**Table (6): Relation of studied parameters with clinicopathological data in piles group.**

|  | **n** | **Circ-SMARCA5** | **Circ-NOL10** | **Circ-LDLRAD3** | **Circ-RHOT1** | **CEA** |
| --- | --- | --- | --- | --- | --- | --- |
| **Sex** |  | 0.38 ± 0.93  0.13 ± 0.14 | 113.8 ± 109.2  132.6 ± 109.7 | 30.21 ± 13.63  42.59 ± 32.03 | 3.45 ± 3.46  3.24 ± 2.21 | 3.30 ± 1.20 |
| **Male** | 6  4 |  |  |  |  |  |
| **Female** |  |  |  |  |  | 3.33 ± 2.48 |
| **U (P)** | U =5.000 (P=0.171) | | U=9.000(P=0.610) | U =11.000 (P=0.914) | U =9.000 (P=0.610) | U =9.000 (P=0.610) |
|  |  | |  |  |  |  |
| **Smoking** |  | 0.13 ± 0.14  0.38 ± 0.93 | 132.6 ± 109.7  113.8 ± 109.2 | 42.59 ± 32.03  30.21 ± 13.63 | 3.24 ± 2.21  3.45 ± 3.46 |  |
| **No**  **Yes** | 4  6 |  |  |  |  | 3.33 ± 2.48  3.30 ± 1.20 |
| **U (P)** | U =5.000 (P=0.171) | | U =9.000 (P=0.610) | U =11.000 (P=0.914) | U =9.000 (P=0.610) | U =9.000 (P=0.610) |
|  |  | |  |  |  |  |
| **Blood Pressure** | | 0.04 ± 0.10  0.51 ± 0.99 | 133.2 ± 113.6  109.4 ± 104.3 | 29.58 ± 8.79  40.74 ± 30.64 | 4.49 ± 3.43  2.24 ± 1.93 | 3.76 ± 2.16  2.87 ± 1.11 |
| **No**  **Yes** | 5  5 |  |  |  |  |  |
| **U (P)** | U =5.000 (P=0.151) | | U =11.000 (P=0.841) | U =10.000 (P=0.690) | U =8.000 (P=0.421) | U =9.000 (P=0.548) |
|  |  | |  |  |  |  |
| **Types** |  |  | 71.77 ± 66.86  154.3 ± 115.6 |  |  |  |
| **Internal**  **External** | 4  6 | 0.12 ± 0.14  0.38 ± 0.92 |  | 45.65 ± 30.05  28.17 ± 13.66 | 3.12 ± 2.18  3.53 ± 3.46 | 3.90 ± 2.53  2.92 ± 0.91 |
| **U (P)** | U=11.00 (P=0.914) | | U=8.00 (P=0.476) | U=9.00 (P=0.610) | U=12.00 (P=1.000) | U=11.00 (P=0.914) |
|  |  | |  |  |  |  |

The results were expressed mean ± S.D.

U: Mann Whitney test

P: P value for comparing between different categories.
